# TLE1 as a key regulator of osimertinib resistance and EMT in lung adenocarcinoma: implications for prognosis and immunotherapy response

**DOI:** 10.1186/s41065-026-00690-x

**Published:** 2026-05-28

**Authors:** Na Chen, Tianlin Wang, Yi Li, Ruixia Zhang, Xiduan Wei, Zhenglu Wang

**Affiliations:** 1 Biological Sample Resource Sharing Center, Tianjin First Central Hospital, Baoshan West Road, Tianjin, China; 2 Institute of Transplantation Medicine, Nankai University, Tianjin, China; 3 School of Pharmacy, China Pharmaceutical University, Nanjing, China; 4 Deptartment of Pharmacy, Tianjin First Central Hospital, Tianjin, China; 5 School of Pharmaceutical Sciences, Tsinghua University, No.30 Shuangqing Road, Haidian District, Beijing, China

**Keywords:** Lung adenocarcinoma, Osimertinib tolerance, TLE1, Immune microenvironment, Prognosis

## Abstract

**Background:**

Acquired resistance to epidermal growth factor receptor-tyrosine kinase inhibitors (EGFR-TKIs) poses a considerable challenge for the long-term treatment of non-small cell lung cancer (NSCLC). This study aims to identify a novel biomarker associated with osimertinib resistance and explore its role in EGFR-TKI resistance in lung adenocarcinoma (LUAD).

**Methods:**

Comprehensive bioinformatics analysis were done using transcriptomic data from the TCGA and GEO databases to identify genes associated with osimertinib tolerance. Candidate genes were further screened against a histone modification-related gene set from MSigDB, leading to the identification of TLE1. The hub gene, TLE1, was further evaluated through survival analysis, GSEA and CIBERSORT analyses. Loss-of-function experiments performed *in vitro* were utilized to assess the impact of TLE1 on osimertinib resistance in LUAD.

**Results:**

TLE1 was of particularly noteworthy, exhibiting elevated expression in LUAD, especially in stage IV patients, and was associated with poor prognosis. GSEA results showed that mTOR, VEGF and HIF-1 signaling pathways were notably activated in the high TLE1 expression group. CIBERSORT analysis markedly elevated levels of regulatory T cells (Tregs) in the TLE1 high expression group. Additionally, epithelial-mesenchymal transition (EMT) scores were significantly elevated in osimertinib tolerant groups and exhibited a positive correlation with TLE1 expression. Furthermore, TLE1 expression was significantly higher in osimertinib-resistant PC9 cells compared to parental PC9 cells. Downregulation of TLE1 enhanced the sensitivity of osimertinib-resistant PC9 cells to osimertinib.

**Conclusions:**

TLE1 was identified as a critical gene associated with osimertinib tolerance, influencing LUAD progression, potentially serving as a novel therapeutic target.

**Clinical trial number:**

Not applicable.

**Supplementary Information:**

The online version contains supplementary material available at 10.1186/s41065-026-00690-x.

## Introduction

Lung cancer, also known as pulmonary carcinoma, originates from malignancies that develop in the bronchial mucosa or glandular tissues of the lungs [1]. Non-small cell lung cancer (NSCLC) represents 80–85% of all lung cancer cases and includes subtypes such as lung adenocarcinoma (LUAD), lung squamous cell carcinoma, and large cell lung carcinoma [2, 3]. LUAD, a predominant NSCLC subtype, poses a major public health challenge due to its high incidence and mortality [4]. Currently, surgical resection remains the standard treatment for early-stage LUAD, while chemotherapy is extensively utilized in patients with advanced-stage LUAD. The advent of epidermal growth factor receptor tyrosine kinase inhibitors (EGFR-TKIs) has transformed treatment paradigms for EGFR-mutant LUAD [5, 6]. First and second-generation EGFR-TKIs are primarily utilized for individuals with EGFR-sensitizing mutations, such as exon 19 deletions and L858R mutations [7, 8]. The clinical adoption of EGFR-TKIs has led to notable improvements in survival rates and treatment outcomes for LUAD patients with EGFR mutations [9, 10]. Consequently, many patients develop acquired resistance within 9 to 14 months of treatment [11]. Osimertinib, a third-generation EGFR-TKI, overcomes T790M-mediated resistance and has demonstrated superior efficacy in the first-line setting [12]. The FLAURA trial reported a median progression-free survival (PFS) of 18.9 months with osimertinib versus 10.2 months with earlier-generation TKIs [13], along with improved central nervous system (CNS) penetration and overall survival (OS). Nevertheless, the inevitable emergence of osimertinib resistance underscores the urgent need to elucidate novel resistance mechanisms.

Emerging evidence highlights the critical role of epigenetic dysregulation in EGFR-TKI resistance. Key findings indicate that mechanisms such as DNA methylation, histone modifications, and chromatin remodeling significantly contribute to the development of acquired resistance to targeted therapies in NSCLC. The activation of TEAD-YAP is significantly enhanced in EGFR-mutant NSCLC patients treated with both EGFR inhibitor (Osimertinib) and MEK inhibitor (Selumetinib), resulting in the development of acquired drug resistance [9, 14]. Fernando et al. have demonstrated, using RNA-seq, ATAC-seq, and CUT&TAG techniques, that the mammalian SWI/SNF chromatin remodeling complex plays a crucial role in mediating osimertinib resistance in EGFR-mutated lung cancer by modulating NRF2-related signaling pathways [15]. The loss of DNA methylation dioxygenase TET2 has been shown to facilitate EGFR-TKI resistance in NSCLC by relieving transcriptional repression of the TNF/NF-κB signaling pathway [16]. Overall, epigenetic modifications, such as DNA methylation, non-coding RNA regulation, and chromatin remodeling, have been extensively studied in the context of EGFR-TKI resistance in NSCLC. However, the role of specific genes derived from histone modification-related gene sets in EGFR-TKI resistance remains underexplored.

The present study aims to screen for key genes from a histone modification-related gene set that are associated with EGFR-TKI osimertinib resistance, through integrated bioinformatics analysis and experimental validation. Our investigation revealed that TLE1 (transducin-like enhancer of split 1), a transcriptional corepressor of the Groucho/TLE family [17], exhibits significant dysregulation in osimertinib-resistant LUAD cells. Emerging as a critical oncogene, TLE1 promotes LUAD progression and chemoresistance [18, 19]. Notably, TLE1 has been reported to function as an epigenetic regulator by recruiting histone deacetylases (HDACs) and other chromatin modifiers, thereby establishing repressive chromatin states at target gene loci [19]. Additionally, transgenic overexpression of Grg1 (the murine TLE1 homolog) has been found to induce LUAD-like tumors [20], suggesting its tumorigenic effect in LUAD. Additionally, elevated levels of TLE1 expression have been linked to poor prognosis of LUAD patients [21]. Importantly, prior research has revealed that TLE1 overexpression promotes gefitinib resistance in LUAD cells through inhibition of E-cadherin [18], further supporting its role in EGFR-TKI resistance. These findings collectively highlight the critical role of TLE1 in LUAD progression, prognosis and EGFR-TKI resistance.

In summary, our study demonstrates the upregulation of TLE1 expression in osimertinib-resistant LUAD cell lines, along with a partial restoration of osimertinib sensitivity following TLE1 inhibition. These findings establish TLE1 as a critical mediator of resistance and a promising therapeutic target. Meanwhile, this study may provide valuable insights for developing novel combination strategies to overcome treatment resistance and improve clinical outcomes in EGFR-mutant LUAD patients.

## Materials and methods

### Data sources

The mRNA expression profiling data, including 59 normal lung tissues and 526 LUAD samples, and the corresponding clinical information were obtained from The Cancer Genome Atlas (TCGA) database (https://portal.gdc.cancer.gov/). At the same time, the maf file of LUAD for 516 LUAD samples was downloaded for subsequent analysis. In addition, we also downloaded the datasets GSE222820 (Illumina NovaSeq 6000) and GSE253742 (Illumina NovaSeq 6000), from the Gene Expression Omnibus (GEO database; https://www.ncbi.nlm.nih.gov/geo/). There were 3 osimertinib-tolerant samples and 3 normal samples in the GSE222820 dataset. The GSE253742 dataset consisted of 4 osimertinib-treated samples with 4 normal samples.

miRTarBase (http://mirtarbase.mbc.nctu.edu.tw/index.html) database was used to predict the target genes of miRNA-mRNA interaction pairs. Table S1 presented that 718 histone modification-associated genes was retrieved and downloaded from the MSigDB database (https://www.gsea-msigdb.org/gsea/msigdb).

### Identification of differentially expressed genes (DEGs)

For differential analysis, we utilized the “limma” package (version 3.5.2) in R language to identify DEGs between two groups [22]. For each gene, limma calculated the log2 fold change (log2FC) and generated adjusted p-values using the Benjamini-Hochberg false discovery rate (FDR) correction to account for multiple hypothesis testing. Genes that meet the threshold of |log2FC| > 1 (indicating absolute fold change > 2) along with an FDR-adjusted p-value ≤ 0.05 were deemed statistically significant DEGs.

### Functional enrichment analysis

To evaluate the potential biological functions and pathways of genes, the “clusterProfiler” package in R language was employed for conducting for GO analysis (including Biological Process (BP), Molecular Function (MF) and Cellular Component (CC)) and Kyoto Encyclopedia of Genes and Genomes (KEGG) pathway enrichment analysis [22]. *P* < 0.05 was used as the significant threshold.

### Gene set enrichment analysis

Gene Set Enrichment Analysis (GSEA) was used to evaluate the association of TLE1 expression with known pathways. The LUAD samples in the TCGA dataset were divided into two groups (TLE1 high expression group and TLE1 low expression group) by the median expression level. Then R package “clusterProfiler” was used to conduct GSEA, and the |normalized enrichment score (NES)| > 1 and adjusted *p*-value < 0.05 were used as the significant threshold.

### Protein-protein interaction (PPI) networks

The functional relationship and interactions among proteins were analyzed by using STRING database (https://string-db.org/, version 11.0) [23], and the PPI network was visualized by using Cytoscape (version 3.7.2) [24]. Finally, the Cytohubba plug-in and Maximum neighborhood component (MCC) algorithm in Cytoscape software were carried out to determine and visualize the hub genes in PPI network.

### Epithelial-mesenchymal transition (EMT)-related markers and EMT scores

A total of 16 canonical gene markers of EMT were collected from previous literature [25]. However, only 15 of these markers were presented in the RNA expression data from the TCGA database, including 3 “Epithelial” markers and 15 “Mesenchymal” markers. Complete canonical EMT-related genes were listed in Table S2. Therefore, we ultimately including these 15 genes for subsequent analysis. Gene expression RNA-sequencing z-scores were obtained from TCGA database. EMT scores were derived by calculating the difference between the mean RNA-seq z-scores of 12 mesenchymal marker genes and the mean RNA-seq z-scores of 3 epithelial marker genes for each individual sample.

### Survival analysis

Survival analysis was conducted utilizing the Kaplan-Meier method, with group comparisons assessed via the log-rank test. The overall survival (OS) rates between different cohorts were estimated using the survival (https://CRAN.R-project.org/package=survival) and survminer packages (https://CRAN.R-project.org/package=survminer) in R. A multivariate Cox regression model was used to analyze whether the TLE1 could predict the survival of LUAD patients independently of other factors.

### Immune cell infiltration analysis

In order to assess the composition of different immune cell types in the tumor microenvironment (TME), we calculated the relative proportions and *P*-value of 22 kinds of infiltrating immune cells in each sample by CIBERSORT software [26]. The CIBERSORT software was applied to characterize the composition of infiltrating immune cells by deconvolution algorithm employing the preset 547 barcode genes based on a gene expression matrix. The sum of the proportions of all estimated immune cell types for each sample equaled 1. In addition, the ESTIMATE package (version 1.0.13) in R was used to calculate stromal score, immune score, ESTIMATE score, and tumor purity for different groups.

### Drug sensitivity prediction

The “oncoPredict” R package [27] was used to predict how sensitive patients are to anti-cancer medications, with a lower IC50 value indicating greater sensitivity. The correlation between TLE1 expression and the IC50 value of each drug was analyzed using the Pearson correlation test.

### Cell culture and treatment

The NSCLC cell lines, including PC9 (#JY123) and osimertinib-resistant PC9 (PC9-OR, #JY1068), were purchased from Shanghai Jinyuan Biotechnology Co., Ltd. Both PC9 and PC9-OR cells were cultured in RPMI-1640 medium (Gibco, #11875500) supplemented with 10% fetal bovine serum (FBS; BI, #04-001-1ACS) and 1% penicillin-streptomycin solution (Gibco, #15140122) at 37 °C in a humidified atmosphere containing 5% CO₂. HEK293T cells were cultured in DMEM (Gibco, #11995500) supplemented with 10% FBS and 1% penicillin-streptomycin solution at 37 °C with 5% CO₂.

PC9 and PC9-OR cells were treated with different concentrations (0, 0.001, 0.005, 0.01, 0.1, 0.5, 2.5, 5, 10 µM) of osimertinib (Selleck chemicals, #AZD9291) for 72 h.

### Cell transfection

The pSLenti-U6-shRNA(NC/TLE1)-CMV-EGFP-F2A-Puro-WPRE plasmids and lentiviral packaging vectors (psPAX and pMD2.G) were obtained from Shanghai Obio Technology. The lentivirus was carried out by transfecting the plasmids and the packaging vectors into HEK293T cells using PEI MAX solution (Polysciences, #24765). The viral supernatant was harvested at 24–72 h post-transfection, filtered through a 0.45 μm membrane, and aliquoted for subsequent transfection. Subsequently, PC9 and PC9-OR cells were transduced with the lentiviral particles and selected with puromycin (0.5–2 µg/mL, Yeasen, #60210ES25) for 72 h to establish stable cell lines.

### Cell viability assay

PC9 and PC9-OR cells (2000 cells/well) were plated into 96-well plates overnight. Then, the CellTiter-Glo^®^ luminescent cell viability assay kit (Promega, #G7571) was employed to assess cell viability at 72 h according to the manufacturer’s protocol.

### Real-time quantitative reverse transcription PCR (RT‑qPCR)

Approximately 2.5 × 10^5^cells were lysed in TRIzol reagent (Termo Fisher Scientifc, #15596026CN) for total RNA extraction. Next, cDNA synthesis and quantitative PCR were performed via the PrimeScript™ RT reagent Kit with gDNA Eraser (Takara, #RR047) and TB Green^®^ Premix Ex Taq™ II FAST qPCR (Takara, #CN830), respectively. GAPDH was used as an internal control, and the relative mRNA levels were calculated with 2^−∆∆Ct^ method. The primers used in this study were as follows: human TLE1 forward primer: 5’-GAGTCCCTGGACCGGATTAAA-3’ and reverse primer: 5’-AATACATCACATAGTGCCTCTGC-3’; GAPDH forward primer: 5’-CATCCTGGGCTACACTGAGC-3’ and reverse 5’-AAAGTGGTCGTTGAGGGCAA-3’.

### Western blot

Proteins were separated by sodium dodecyl sulfate-polyacrylamide gel electrophoresis (SDS-PAGE) and transferred onto polyvinylidene fluoride membranes. Afterward, the membranes were blocked with Protein Free Rapid Blocking Buffer 1x (Shanghai Epizyme Biomedical Technology, #PS108P) at room temperature for 1 h and incubated with mouse anti-human TLE1 (1:500; Santa Cruz Biotechnology, #SC137097) or rabbit-anti-human β-actin (1:10000; Affinity, # AF7018) primary antibodies at 4℃ overnight. Then, membranes were incubated with HRP-conjugated goat anti-rabbit or mouse IgG (1:10000; Affinity, #S0001 or #S0002) secondary antibody at room temperature for 1 h. Finally, the blots were visualized using the enhanced chemiluminescence reagents (Shanghai Epizyme Biomedical Technology, #SQ201).

### Colony formation assay

Five hundred cells were seeded in a 6-well plate, and then incubated for 14–21 days. Subsequently, the cells were washed with PBS, fixed with a 4% neutralized formaldehyde solution at room temperature for 20 min, and then stained with crystal violet (Solarbio, #C1062). Finally, the plate was air-dried, and the colonies were captured using a digital camera.

### Statistical analysis

For the bioinformatics analysis, variations in infiltrating immune cells and TLE1 expression among different groups was calculated by using Wilcoxon rank-sum tests. The survival analysis of different groups was performed utilizing the Kaplan-meier (KM) survival analysis with a log-rank test (“Survival” and “Survminer” R packages). *P* < 0.05 was considered as the significant threshold. All statistical analyses were accomplished by R version 3.5.2.

The cellular experiments were conducted independently at least three times, and the results are are presented as mean ± standard deviation. GraphPad Prism V9.0 was applied for statistical analysis. Comparisons between two groups were performed using an unpaired two-tailed Student’s t test. One-way ANOVA was utilized to analyze differences among three groups. *P* < 0.05 was considered statistically significant.

## Results

### Identification of osimertinib resistance-related genes

**Fig. 1:**
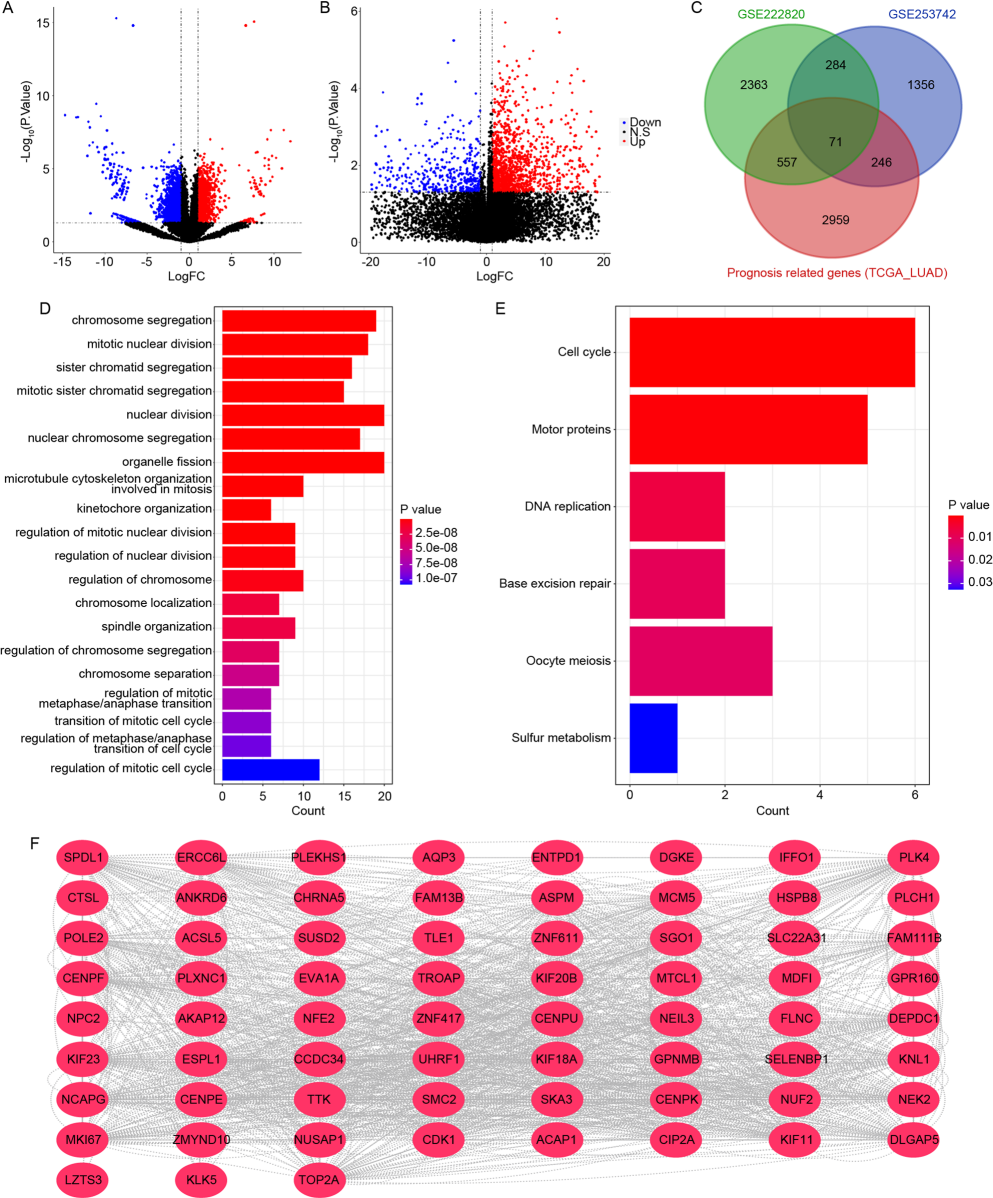
PPI and enrichment analysis of overlapping genes in public databases. **A** Volcano plots shows all differentially expressed genes in GSE222820 dataset between osimertinib-tolerant group and control group. The blue dots represented the down-regulated genes and the red dots represented the up-regulated genes. **B** Volcano plots shows all differentially expressed genes in GSE253742 cohorts between osimertinib-treated group and control group. The blue dots represented the down-regulated genes and the red dots represented the up-regulated genes. **C** Prognostic genes from TCGA_LUAD cohort and differentially expressed genes in GSE222820 and GSE253742 datasets were intersected by a Venn diagram. **D** Bubble chart of the top 20 significantly enriched GO terms. **E** Bubble chart of six significantly enriched KEGG pathways. **F** A PPI network analysis of all the 71 overlapping genes

Firstly, to identify key genes associated with osimertinib, we conducted differential expression analysis using two datasets. In the GSE222820 dataset, which compared osimertinib-tolerant cells (OR) to normal cells (Control), we identified 1,457 upregulated genes and 1,818 downregulated genes (Fig. 1A). Similarly, in the GSE253742 dataset, which comparing osimertinib-treated cells (OR) with normal cells (Control), we detected 1,387 upregulated genes and 570 downregulated genes (Fig. 1B). Furthermore, univariate cox regression analysis of the TCGA-LUAD revealed 3,833 genes significantly associated with prognosis (Table S3). By intersecting the differentially expressed genes from GSE222820 and GSE253742 with these 3,833 prognostic genes, 71 overlapping genes were obtained (Fig. 1C). Then, we performed GO and KEGG enrichment analysis based on these 71 overlapping genes, which were enriched in 242 GO terms (top 20 terms, Fig. 1D), and 6 KEGG pathways (Fig. 1E). All detailed functional enrichment results were summarized in **Table S4**. In addition, to analyze the interactions of the 71 overlapping genes, a PPI network containing 67 nodes and 1302 edges was constructed using STRING online database (Fig. 1F).

### Identification of the key gene TLE1 in LUAD

**Fig. 2:**
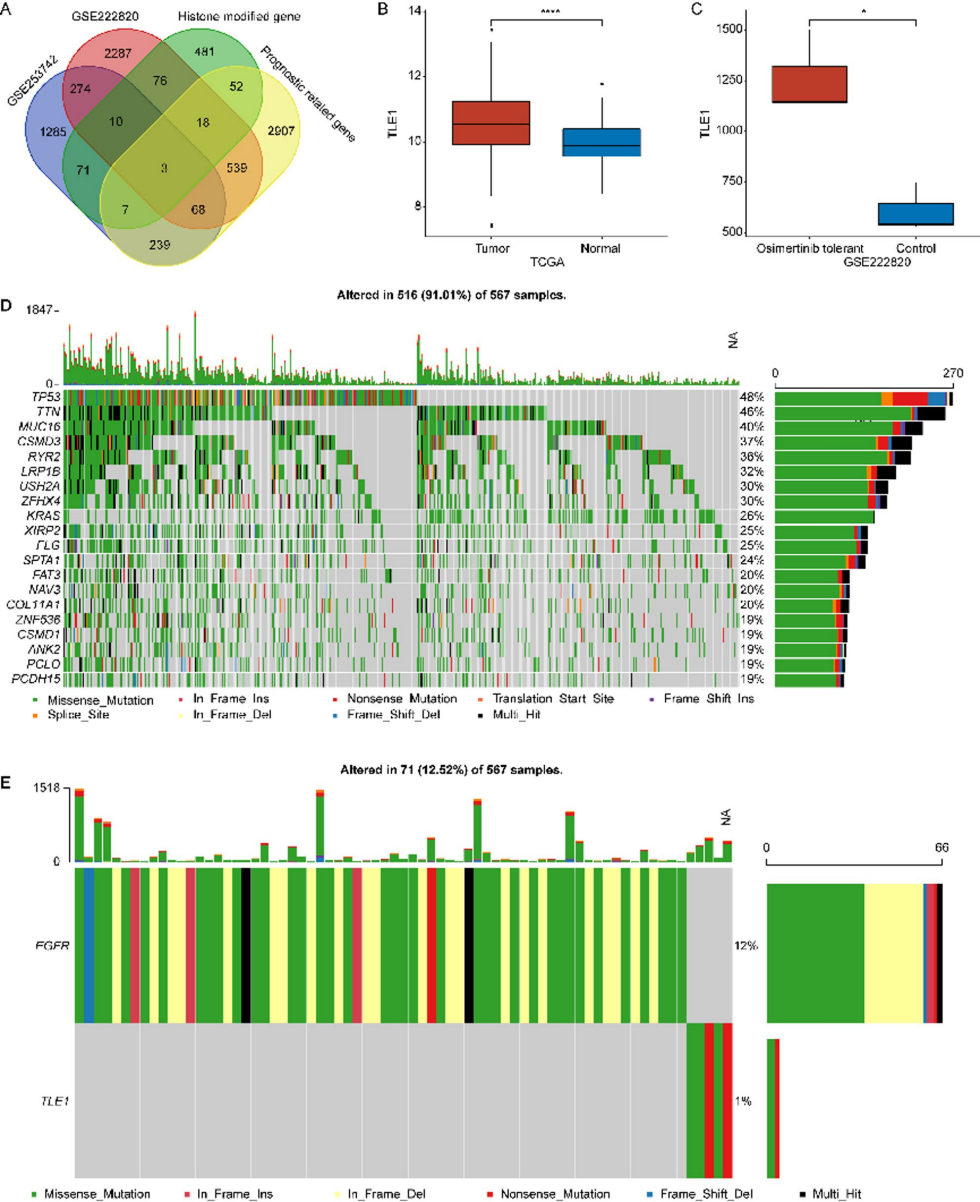
Identification of TLE1 as a key gene and its expression and mutation profile in LUAD.** A** The Venn diagram illustrates the intersections among differentially expressed genes from GSE222820 and GSE253742 datasets, prognosis-associated genes from TCGA-LUAD cohort and 718 histone modification-related genes from MSigDB database. A total of 3 common genes were identified. **B** TLE1 was upregulated in LUAD tissues than in normal lung tissues in the TCGA_LUAD cohort. **C** TLE1 expression in osimertinib-tolerant PC9 cells was significantly higher than that in control cells in the GSE222820 dataset. **D** Depiction of the mutation landscape of LUAD samples in the TCGA_LUAD cohort. **E** Depiction of the mutation landscape of EGFR and TLE1 in the TCGA-LUAD cohort

Subsequently, 718 histone modification-related genes were downloaded from the MSigDB database (https://www.gsea-msigdb.org/gsea/msigdb) and intersected with the 71 osimertinib resistance-related genes identified above, resulting in the identification of three overlapping genes: UHRF1, TLE1, and CDK1 (Fig. 2A). Among them, TLE1, a member of the vertebrate Gro/TLE family of proteins, is a co-repressor involved in diverse developmental functions [28–30]. TLE1 has been implicated in the pathogenesis of several malignancies, with emerging evidence indicating its aberrant expression or upregulation in various human cancers, including synovial sarcoma [31], breast cancer [32], and lung cancer [20]. Given its significant role in cancer pathobiology, we focused on TLE1 in further studies. We first examined TLE1 expression in LUAD using data from the TCGA database. The results indicated a marked increase in TLE1 expression levels within LUAD tissue samples (Fig. 2B). Furthermore, the mRNA levels of TLE1 in osimertinib-tolerant PC9 cells were considerably greater compared to those in the control group (Fig. 2C). Somatic mutation was then analyzed in the TCGA-LUAD dataset by maftools. The top five mutated genes included TP53, TTN, MUC16, CSMD3, and RYR2 (Fig. 2D). Specifically, of the 71 samples with EGFR or TLE1 mutations, we found that 66 samples had EGFR mutations and 5 samples had TLE1 mutations. Interesting, no samples had both EGFR and TLE1 mutations (Fig. 2E).

### Identification of TLE1-related functional information and interaction network

**Fig. 3:**
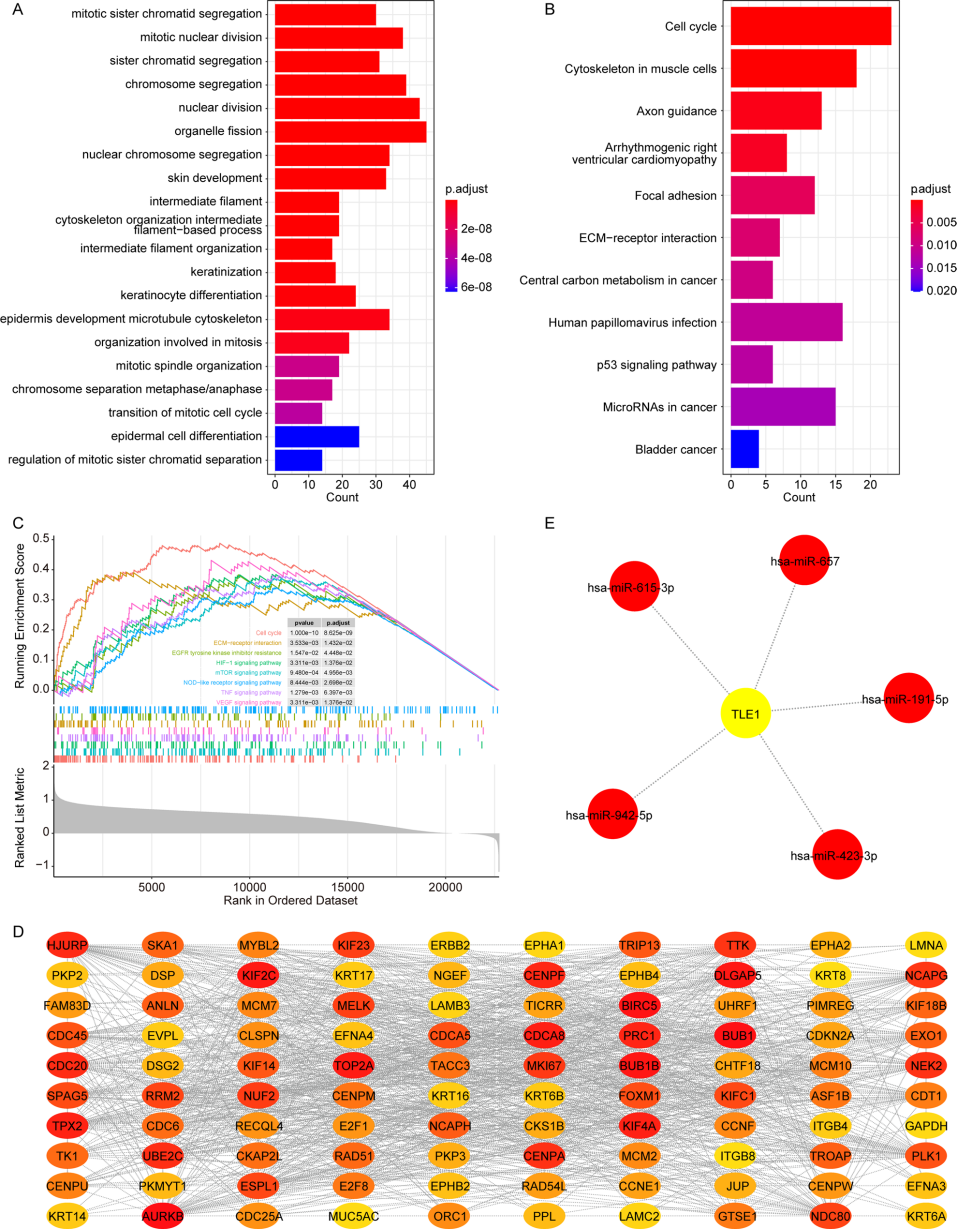
Functional enrichment and PPI network analysis of TLE1-related differentially expressed genes in LUAD. **A** Bar plot showing the top 20 signaling pathways enriched in GO terms. **B** Bar plot showing the all 11 signaling pathways enriched in KEGG terms. **C** The eight enriched signaling pathways between the TLE1 high expression and TLE1 low expression groups with the GSEA analysis. **D** The top 100 hub genes identified from the PPI network. The darker the color, the more significant the gene is. **E** The potential miRNAs targeted by TLE1 were predicted by miRTarBase

Based on the median expression value of TLE1, patients were divided into TLE1 high expression group (TLE1 expression levels greater the median value) and TLE1 low expression group (TLE1 expression levels less than the median value). Subsequently, in TCGA-LUAD cohort, we identified 506 genes with significant differential expression between two groups (Table S5). GO enrichment analysis of these 506 genes revealed that they were mainly related to 243 GO terms, including mitotic sister chromatid segregation, nuclear division, and keratinocyte differentiation (Fig. 3A, Table S6). Meanwhile, KEGG pathway analysis showed that these 506 genes were primarily enriched in 11 KEGG pathways, such as cell cycle, ECM-receptor interaction, p53 signaling pathway, and MicroRNAs in cancer (Fig. 3B, Table S6). Additionally, we also conducted GSEA enrichment analysis comparing the TLE1 high expression and low expression groups. The findings showed multiple cell proliferation-related pathway, such as cell cycle, mTOR signaling pathway, HIF-1 signaling pathway, TNF signaling pathway, VEGF signaling pathway, ECM-receptor interaction, EGFR tyrosine kinase inhibitor resistance and NOD-like receptor signaling pathway, were significantly enriched in TLE1 high expression group (Fig. 3C).

To investigate the interactions among TLE1-related genes, PPI networks were generated using the STRING database and visualized with Cytoscape software, consisting of 240 nodes and 2622 edges. Next, utilizing multi-network clustering (MNC) approaches, the top 100 core nodes were subsequently identified (Fig. 3D, Table S7). Moreover, regarding the TLE1 gene, we utilized the miRTarBase database to predict potential miRNAs that may regulate TLE1, identifying five specific miRNAs: miR-615-3p, miR-657, miR-191-5p, miR-423-3p, and miR-942-5p (Fig. 3E).

### Characterization of immune cell infiltration between the TLE1 high and low expression groups

**Fig. 4:**
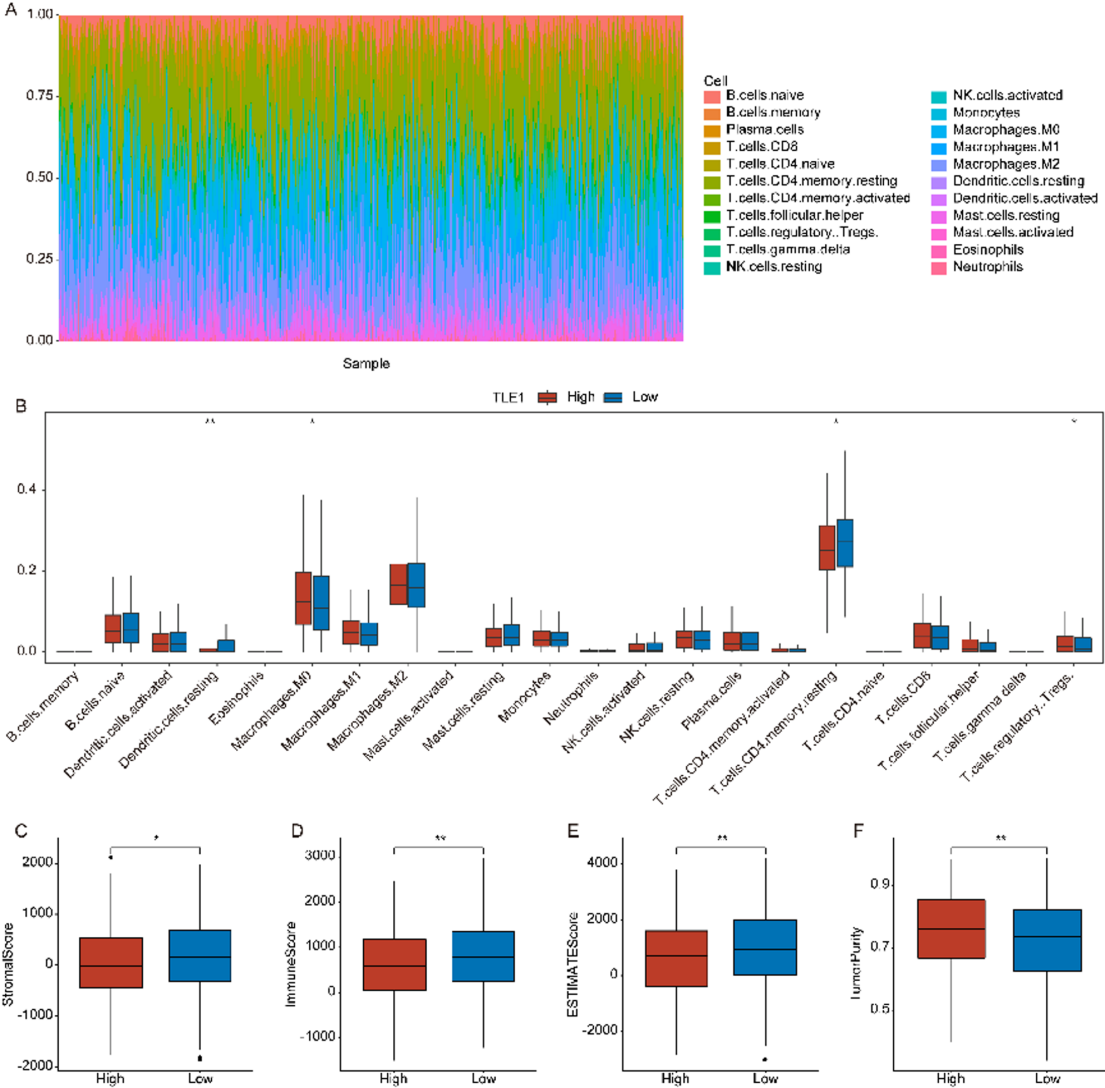
Immune infiltration analysis.** A** A bar chart showing the difference in the proportion of 22 kinds of immune infiltration cells in the TME of LUAD in the TCGA_LUAD cohort. **B** Boxplots comparing the proportion of the 22 types of immune infiltration cells in the TME of LUAD between the the TLE1 high and low expression groups. **C-F** Analysis of differences in (**C**) StromalScore, **D** ImmuneScore, **E** EstimateScore, and (**F**) TumorPurity between the two groups. *P < 0.05, **P < 0.01

The proportions of 22 distinct immune lymphocyte populations in LUAD samples within TCGA cohort were estimated through the CIBERSORT algorithm (Fig. 4A). The results revealed that TLE1 high expression group exhibited higher distributions of Macrophages M0, and Tregs compared to the TLE1 low expression group; conversely, a decrease in the infiltration of resting dendritic cells, resting CD4^+^ memory T cells was observed in the TLE1 high expression group (Fig. 4B). Moreover, StromalScore, ImmuneScore, and ESTIMATEScore were obviously lower in the TLE1 high expression group relative to their low expression counterparts (Fig. 4C-E). On the contrary, the TumorPurity was notably elevated in the TLE1 high expression group (Fig. 4F).

### TLE1 was considered to be associated with EMT in LUAD

**Fig. 5:**
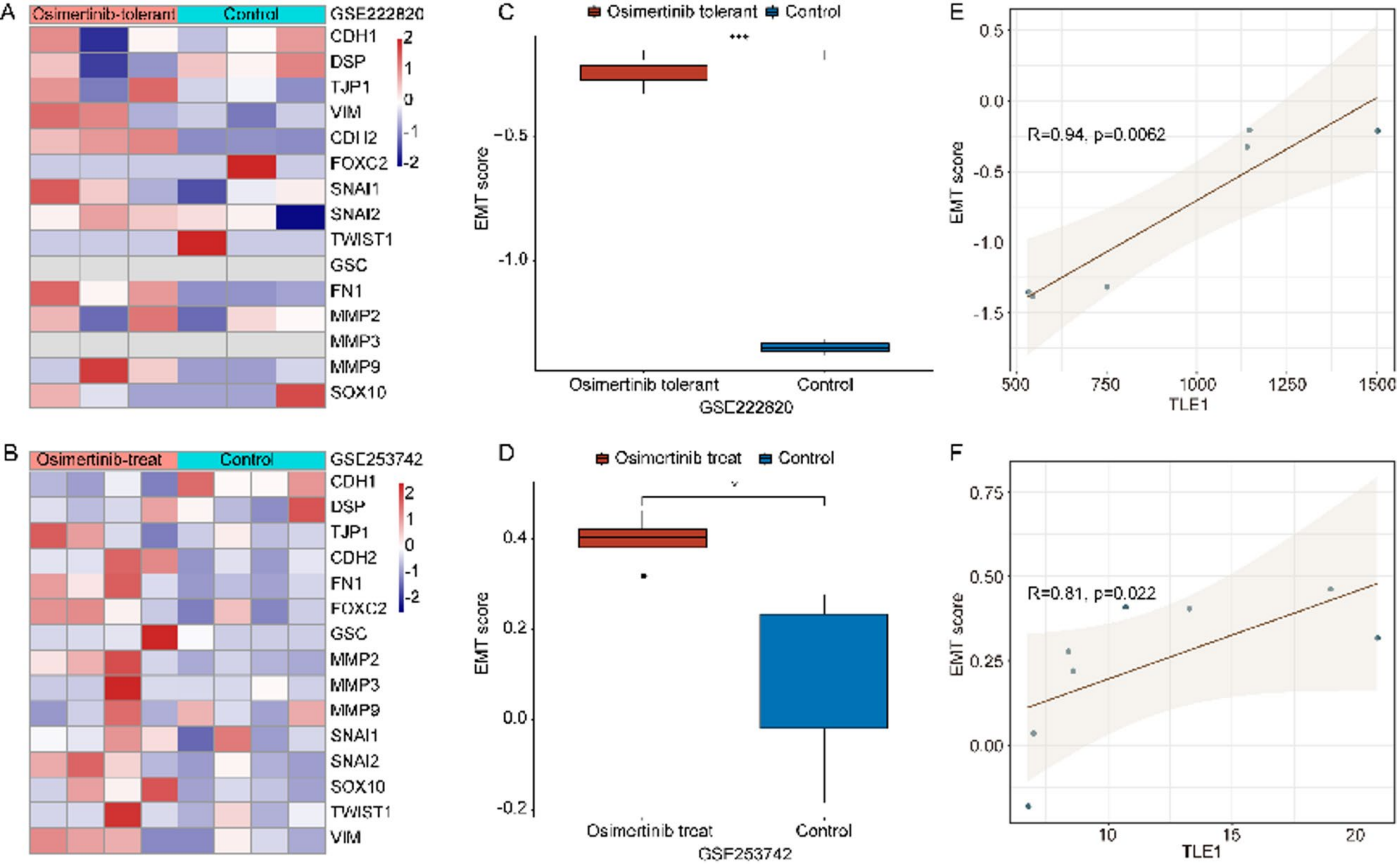
TLE1 was associated with EMT in LUAD by bioinformatics analysis. **A** Heatmap showing the expression patterns of 15 EMT-related markers in osimertinib-tolerance and control groups in the GSE222820 dataset. **B** Heatmap showing the expression patterns of 15 EMT-related markers in osimertinib-treat and control samples in the GSE253742 datasets. **C** Illustration of the relationship between the TLE1 expression and EMT score in the GSE222820 dataset. **D** Depiction of the relationship between TLE1 expression and EMT score in the GSE253742 datasets. **E** Correlation analysis between TLE1 expression and EMT score in the GSE222820 cohort. **F** Correlation analysis between TLE1 expression and EMT score in the GSE253742 cohort

EMT is a well-established mechanism underlying resistance to EGFR-TKIs, as noted in previous studies [33]. In particular, osimertinib-induced EMT has been extensively documented [34]. Tanaka and colleagues demonstrated that osimertinib-resistant cell lines, such as H1975R and ECLC26R, undergo EMT and become susceptible to AURKB inhibition [35]. Therefore, to further investigate the potential relationship between the resistance-related gene TLE1 and EMT, we first selected 15 EMT-related markers and analyzed their expression profiles in two independent datasets, GSE222820 and GSE253742 (Fig. 5A, B). The results indicated a relatively obvious differential expression pattern between osimertinib-tolerant/treated groups and their corresponding control groups. Next, we calculated EMT scores for the samples in both datasets using the ssGSEA method. In both cohorts, the EMT scores were significantly higher in the osimertinib-tolerance/treated groups compared to control groups (Fig. 5C, D), suggesting that osimertinib may promote EMT progression in LUAD. Notably, Pearson correlation analysis identified a strong positive correlation between TLE1 and EMT scores in both datasets (Fig. 5E, F), indicating a potential role for TLE1 in EMT regulation. Further results revealed that TLE1 expression was positively correlated with certain EMT markers, including CDH2 and FN1, in the GSE222820 cohort (Figure S1). Similarly, in the GSE253742 cohort, TLE1 showed strong positive correlations with CDH2 and SOX10 (Figure S2). These bioinformatics findings suggested that TLE1 may play a significant role in the EMT process during LUAD progression, particularly in the context of osimertinib resistance.

### Crucial prognostic value of TLE1 in LUAD

To explore the correlation between TLE1 expression and clinicopathological characteristics, we analyzed its expression across various subgroups based on clinical parameters, including age, gender, smoking status, and disease stage, in the TCGA_LUAD cohort. Our analysis revealed that TLE1 expression was significantly higher in patients with stage IV disease compared to those with stage I or stage III (Figure S3A). However, no statistically significant associations were observed between TLE1 expression and other clinical variables, such as age, gender, or smoking status (Figure S3B-D), suggesting that TLE1 expression is not influenced by these factors.

**Fig. 6:**
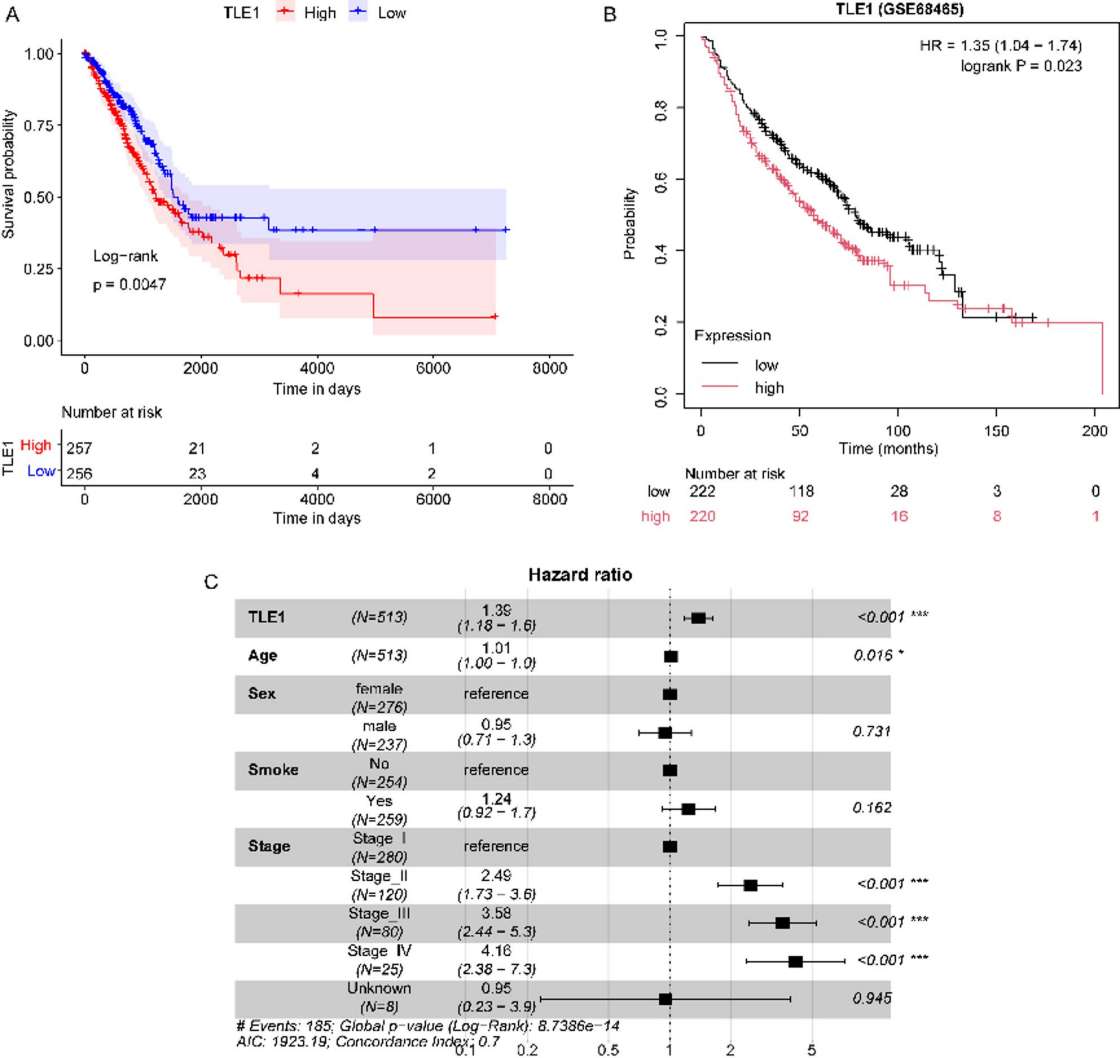
Survival analyses.** A** Kaplan-Meier survival analysis comparing overall survival between TLE1 high and low expression groups in TCGA_LUAD cohort (Log-rank test). **B** Association between TLE1 mRNA expression and prognosis in LUAD based on the GSE68465 dataset from the KM-plotter database (Log-rank test). **C** Forest plots of multivariate Cox regression incorporating TLE1 and clinical factors

Next, we evaluated the prognostic value of TLE1 in LUAD. Survival analysis demonstrated that patients in the TLE1 high expression group exhibited a remarkably poorer prognosis compared to those in the low-expression group (Fig. 6A). This finding was further validated using the KM-plotter database (https://www.kmplot.com/analysis/), where elevated TLE1 expression was similarly associated with worse survival outcomes in the GSE68465 dataset (Fig. 6B).

To further determine whether TLE1 serves as an independent prognostic factor, a multivariate Cox regression analysis was performed, incorporating age, gender, smoking status, and stage as covariates. The analysis confirmed that high TLE1 expression remained an independent predictor of poor prognosis (Fig. 6C). In summary, these findings suggested that TLE1 was not only associated with advanced disease stages but also served as a significant prognostic biomarker in LUAD, independently correlating with worse survival outcomes.

Given that osimertinib is specifically indicated for EGFR-mutant NSCLC, we performed stratification analyses based on EGFR mutation status in the TCGA-LUAD cohort to determine whether the prognostic value of TLE1 is dependent on EGFR mutation status. The results showed that high TLE1 expression was associated with significantly worse overall survival in the EGFR-mutant subgroup (*P* < 0.05; Figure S4A), whereas no significant difference in survival was observed between the TLE1-high and TLE1-low expression groups in the EGFR-wildtype subgroup (*P* > 0.05; Figure S4B). These findings indicate that the prognostic value of TLE1 is specific to EGFR-mutant LUAD patients.

### Drug sensitivity analysis

**Fig. 7:**
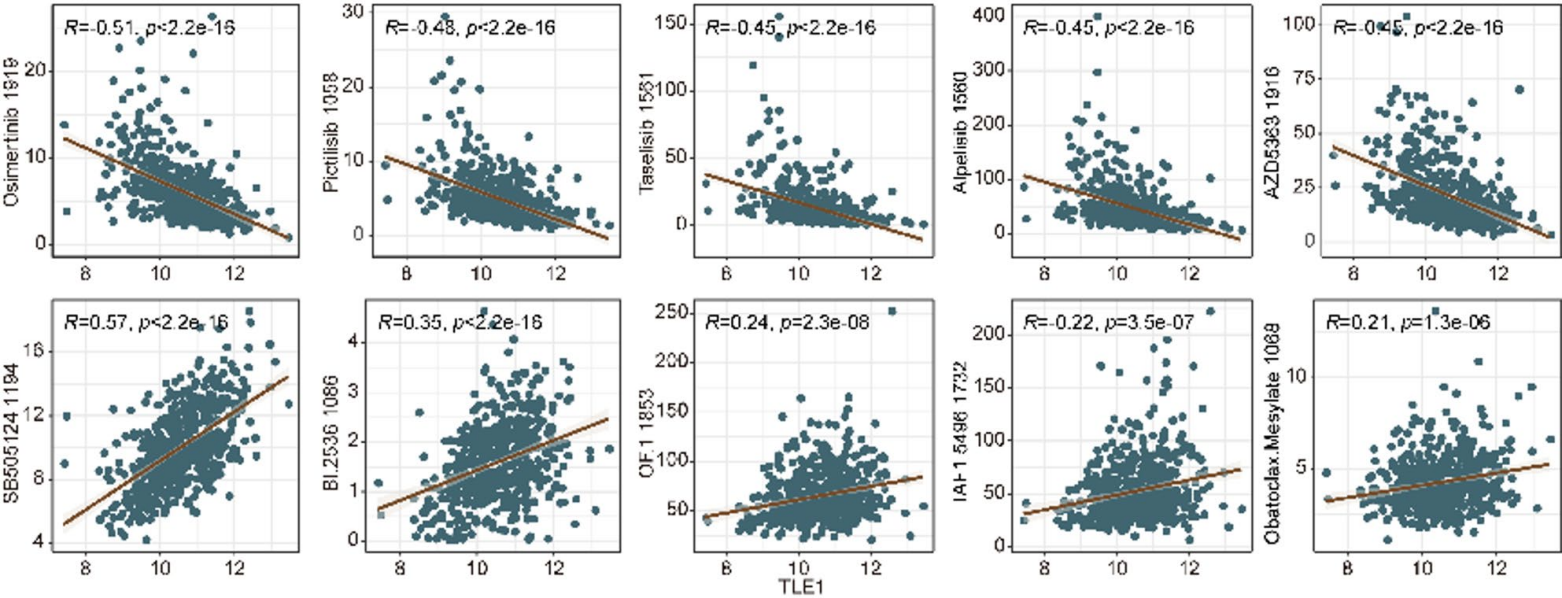
Correlation analysis between TLE1 expression and drug IC50. Correlation of TLE1 expression with the drug sensitivity (IC50 value) to ten drugs

As illustrated in Fig. 7, we conducted an analysis to assess the correlation between TLE1 expression and the sensitivity of various chemotherapeutic agents. The results revealed a significant negative correlation between TLE1 expression and the sensitivity (IC50) of 120 drugs, including Osimertinib_1919, indicating that patients exhibiting higher TLE1 levels may be more sensitive to these treatments (Table S8). Conversely, a notable positive correlation was observed between TLE1 and the sensitivity (IC50) of 20 drugs, such as SB505124_1194 (Table S8). We specifically highlighted the top five drugs with the highest absolute correlation coefficients (Fig. 7). These findings underscore the potential of TLE1 as a predictive biomarker for drug sensitivity in LUAD treatment.

### Downregulation of TLE1 enhanced the sensitivity of PC9-OR cells to osimertinib

**Fig. 8:**
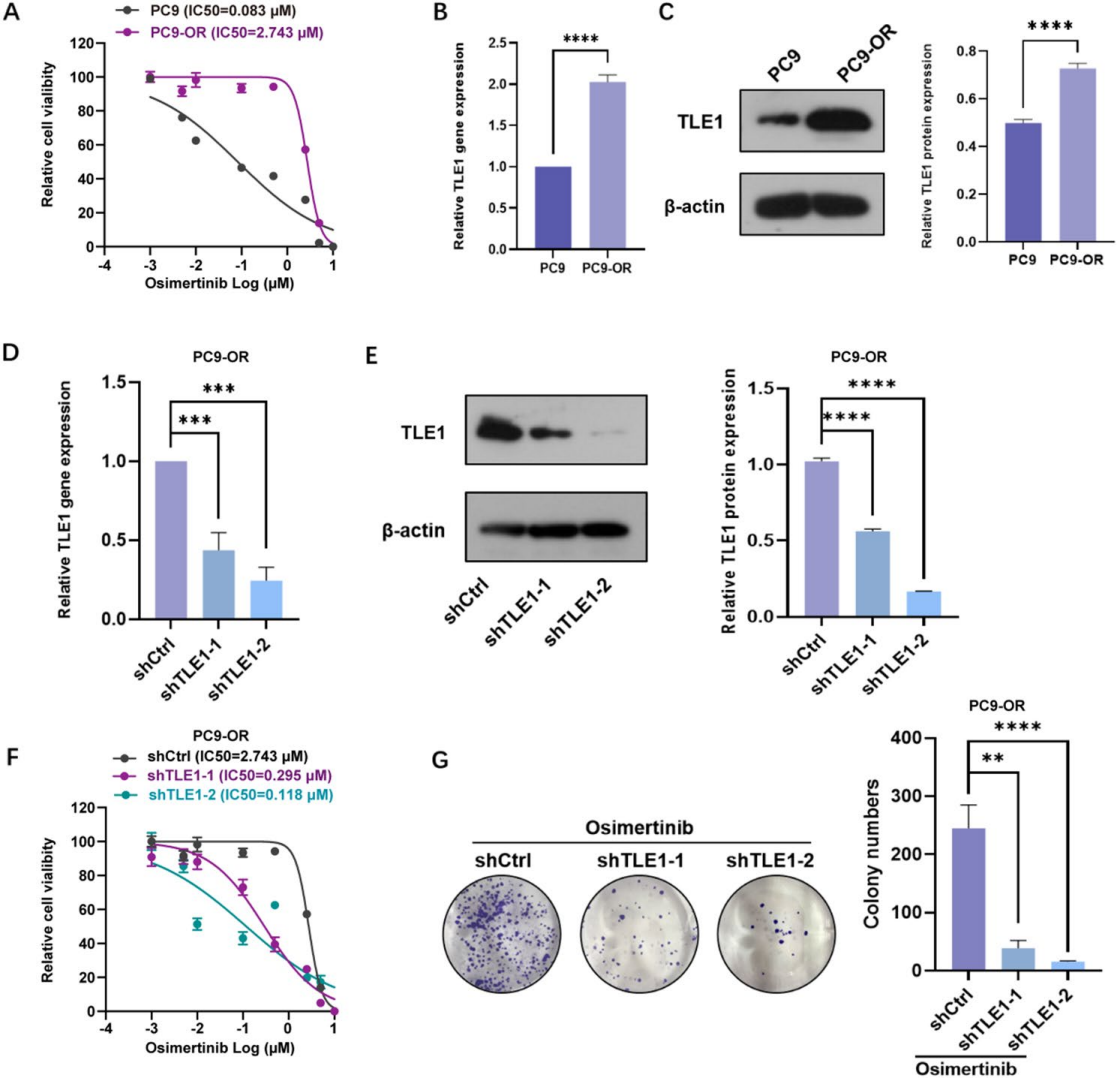
Downregulation of TLE1 enhanced the sensitivity of PC9-OR cells to osimertinib.** A** PC9 and PC9-OR cells were treated with different concentrations (0, 0.001, 0.005, 0.01, 0.1, 0.5, 2.5, 5, 10 µM) of osimertinib for 72 h. CCK-8 assay was employed to assess cell viability. **B** RT-qPCR and **(C)** western blot assays were conducted to evaluate TLE1 levels in PC9 and PC9-OR cells. **D**,** E** PC9-OR cells were transfected with shCtrl, shTLE1-1, shTLE1-2. RT-qPCR and western blot assays were conducted to evaluate TLE1 levels in transfected cells. **F**,** G** PC9-OR cells were transfected with shCtrl, shTLE1-1, shTLE1-2, followed by osimertinib treatment. CCK-8 and colony formation assays were employed to assess cell viability and proliferation. **P<0.01, ***P < 0.001, ****P < 0.0001

To determine whether TLE1 is dysregulated in osimertinib-resistant NSCLC cells, the parental PC9 and osimertinib-resistant PC9 (PC9-OR) cells were utilized. As shown in Fig. 8A, the IC50 value of osimertinib for parental PC9 cells was 0.083 µM, whereas the IC50 value for PC9-OR cells was 2.743 µM. This indicates that PC9-OR cells exhibit resistance to osimertinib compared to parental PC9 cells. Meanwhile, the results of RT-qPCR and western blot assays revealed a significant increase in both mRNA and protein levels of TLE1 in PC9-OR cells, when contrasted with PC9 cells (Fig. 8B and C).

To further investigate the potential role of TLE1 in modulating osimertinib sensitivity in PC9-OR cells, we inhibited TLE1 expression by lentiviral-mediated TLE1 shRNA knockdown (shTLE1-1 and shTLE1-2) (Fig. 8D and E). Furthermore, the IC50 values of osimertinib on PC9-OR cells transfected with shTLE1-1 and shTLE1-2 are 0.295 µM and 0.118 µM, respectively, while the IC50 value for PC9-OR cells transfected with the shCtrl group is 2.743 µM (Fig. 8F). These results indicate that silencing TLE1 leads to significantly increased sensitivity to osimertinib treatment. Meanwhile, the results of colony formation assay showed that TLE1 downregulation potentiated the anti-proliferative effect of osimertinib (Fig. 8G). Collectively, downregulation of TLE1 could enhance the sensitivity of PC9-OR cells to osimertinib.

## Discussion

In recent years, multiple prognostic signatures and biomarkers have been reported in various cancers. For example, a glycosylation-related prognostic model comprising IGHA2, SLC35A2, and BST2 was shown to potentially predict overall survival and immunotherapy response in breast cancer [36]. Similarly, a disulfidptosis-related signature demonstrated strong prognostic value across multiple cancer types [37]. In head and neck squamous cell carcinoma, PAK2 was identified to be associated with malignant phenotypes such as metabolic reprogramming and tumor metastasis, and its high expression was closely correlated with poor patient prognosis [38]. Distinct from the above studies, our research focuses on the clinical challenge of EGFR-TKI resistance and identifies TLE1 from a histone modification-related gene set as a potential biomarker associated with osimertinib resistance in LUAD. Notably, TLE1 not only serves as a potential prognostic indicator for LUAD but also plays a role in regulating osimertinib tolerance. Collectively, TLE1 may serve as a potential biomarker and and therapeutic target associated with osimertinib resistance in EGFR-mutant LUAD patients.

A previous study demonstrated that TLE1 enhances gefitinib resistance in lung cancer [18], supporting its potential role in EGFR-TKI resistance. Consistent with these findings, our results revealed that TLE1 expression was notably elevated in osimertinib-resistant PC9 cells compared to parental PC9 cells. Significantly, downregulation of TLE1 could enhance the sensitivity of osimertinib-resistant PC9 cells to osimertinib, suggesting a close relationship between TLE1 and osimertinib resistance in LUAD. Furthermore, the findings from the GSEA analysis revealed a significant enrichment of the EGFR tyrosine kinase inhibitor resistance, HIF-1, VEGF, and mTOR signaling pathways in the TLE1 high expression group, suggesting a potential linked between TLE1 and these pathways. Both the HIF-1/VEGF and mTOR pathways are integral to cancer development [39, 40]. Notably, these pathways are also linked to resistance against EGFR-targeted therapies [41, 42]. Blocking HIF-1 has been shown to reverse the acquired resistance of the EGFR-mutant lung cancer cell line to gefitinib [42]. Additionally, mTOR functions as a downstream target of EGFR; any dysregulation within the EGFR/AKT/mTOR signaling axis fosters tumor development and contributes to treatment resistance [43, 44]. These findings suggest that TLE1 may contribute to EGFR-TKI resistance (e.g., osimertinib resistance) in LUAD by regulating the HIF-1, VEGF, and mTOR signaling pathways. However, the precise underlying mechanisms require further investigation in future studies.

Increasing evidence indicates that cancer drug resistance is frequently associated with EMT across various cancers, including pancreatic [45], bladder [46], and breast cancers [47]. In our study, EMT scores notably elevated in osimertinib-tolerant groups compared to the control group. Moreover, TLE1 demonstrated a strong positive correlation with EMT scores. These results showed a potential link among TLE1, EMT and chemoresistance. Mechanistically, TLE1 functions as a transcriptional repressor protein that regulates cell growth [48]. Yao et al. found that TLE1 promotes EMT in lung cancer through recruiting histone deacetylase (HDAC) to the promoter of E-cadherin, thereby suppressing its transcription [19]. Furthermore, Seo et al. reported that TLE1 knockdown suppresses the expression of Bcl-2 in synovial sarcoma cells, suggesting that Bcl-2 may be a potential downstream target influenced by TLE1 signaling [31]. Interestingly, Bcl-2 is known to facilitate Twist1 nuclear translocation, which regulates the expression of multiple genes to induce EMT [49]. Collectively, these findings suggest that TLE1 may modulate EMT both directly (e.g. via transcriptional repression) and indirectly (e.g. through Bcl-2/Twist1 axis regulation). Furthermore, the EMT process is also influenced by multiple signaling pathways, including AKT/mTOR, VEGF, and HIF-1 [50–52]. The mTOR complexes play a role in regulating EMT by affecting the cell’s actin cytoskeleton through phosphorylation of PKC and Akt [53]. Similarly, both VEGF and HIF-1 have been shown to promote EMT [50, 52]. Based on these findings, we propose that TLE1 may promote EMT in LUAD by modulating the HIF-1, VEGF, and mTOR signaling pathways, although the exact mechanisms require further investigation. Given the established role of EMT in tumor metastasis and chemotherapy resistance [54, 55], we speculate that TLE1-mediated EMT may also contribute to EGFR-TKI resistance. Notably, we propose that the role of TLE1 in resistance primarily depends on the upregulation of its expression levels rather than gene mutations. In the TCGA-LUAD cohort, EGFR mutations and TLE1 mutations were mutually exclusive (66 cases vs. 5 cases, with no coexistence) (Fig. 2), suggesting that the aberrant activation of TLE1 in the context of EGFR mutations does not rely on genetic mutations. Our experiments further confirmed that TLE1 expression was significantly upregulated in osimertinib-resistant PC9-OR cells, and knockdown of TLE1 restored drug sensitivity (Fig. 8). Therefore, these findings suggest that TLE1 may drive osimertinib resistance primarily through overexpression rather than mutation.

Recently, tumor-infiltrating immune cells have emerged as crucial elements in the emergence of resistance to cancer therapies [56]. The presence of Tregs within the TME correlates with unfavorable outcomes and resistance to treatments [57]. Evidence indicates that cancer patients exhibiting higher Treg infiltration tend to have worse prognoses and lower survival rates [58, 59]. A heightened presence of Tregs is associated with an immunosuppressive TME [58]. Additionally, Wang et al. revealed that CCL20, derived from colorectal cancer cells, recruits Tregs, thereby promoting chemoresistance [60]. Our study revealed significantly elevated levels of Tregs in the TLE1-high expression group, suggesting Tregs may be involved in TLE1-mediated osimertinib resistance—a relationship requiring further investigation. Additionally, our results also showed that patients with TLE1 high expression presented a significantly worse prognosis. Together, these collective findings indicate that increased Treg infiltration may contribute to the poor prognosis observed in the TLE1-high expression group.

Several limitations of this study should be acknowledged. First, although we observed significantly increased infiltration of Tregs and M0 macrophages in the TLE1-high expression group, the TME is a complex ecosystem comprising various components, such as tumor-associated macrophages, cancer-associated fibroblasts and cytokines, which collectively shape an immunosuppressive microenvironment and thereby influence the efficacy of immunotherapy [61, 62]. In this study, we only preliminarily explored the relationship between TLE1 and immune cell infiltration using the CIBERSORT algorithm, and have not comprehensively evaluated its interactions with various immune components. In the future, LUAD-related scRNA-seq datasets could be employed to systematically dissect the associations of TLE1 expression with immune cell infiltration and cell-cell communication at single-cell resolution, thereby providing a deeper understanding of the role of TLE1 in TME immune regulation, and potentially informing immunotherapeutic strategies for TLE1-mediated resistance. Second, although we have validated the prognostic value of TLE1 in the EGFR-mutant subgroup utilizing the TCGA_LUAD cohort, several limitations should be acknowledged. The number of EGFR-mutant samples in TCGA-LUAD is limited (approximately 12%), which may affect statistical power. Additionally, the TCGA database lacks detailed treatment history and duration of resistance for patients receiving osimertinib therapy. Therefore, the direct causal relationship between TLE1 and osimertinib resistance still requires further validation in preclinical models and prospective cohorts. Third, this study has not experimentally validated whether TLE1 influences osimertinib resistance through the regulation of histone modifications, which represents an important direction for future research. Furthermore, our current analysis of the association between TLE1 and HIF-1/mTOR pathways is based solely on public datasets without experimental validation. Future studies should include rescue experiments in TLE1-knockdown PC9-OR cells (via activation of HIF-1α or mTOR) to determine whether TLE1 functions upstream of these pathways, as well as combination treatments with osimertinib and HIF-1α/mTOR inhibitors in TLE1-overexpressing cells to assess restoration of drug sensitivity. Finally, the present study only performed in vitro experiments demonstrating that TLE1 knockdown suppresses osimertinib resistance. Future studies should establish in vivo xenograft models to confirm that TLE1 knockdown enhances osimertinib sensitivity in mice.

## Conclusions

In summary, our findings establish TLE1 as a critical prognostic biomarker in LUAD, with elevated expression correlating with advanced disease progression and diminished survival. Notably, the strong association between TLE1 osimertinib resistance positions it as a potential therapeutic target. Mechanistically, TLE1 appears to drive EMT, revealing potential avenues for enhancing LUAD treatment strategies. However, further investigation is required to translate these insights into clinically viable TLE1-targeted therapies aimed at overcoming drug resistance. 

## Supplementary Information

Supplementary Material 1. Figure S1 Correlation analysis of TLE1 expression level and 15 EMT-related markers in the GSE222820 cohort.

Supplementary Material 2. Figure S2 Correlation analysis of TLE1 expression level and 15 EMT-related markers in the GSE253742 cohort.

Supplementary Material 3. Figure S3 Clinicopathological parameter relevance analysis.** A-D** Box diagram illustrate the relationships between TLE1 expression and various clinical pathological features: Stage (**A**), Age (**B**), Gender (**C**) and Smoking status (**D**) in the TCGA_LUAD cohort.

Supplementary Material 4. Figure S4 Kaplan-Meier survival analysis of TLE1 expression in LUAD patients stratified by EGFR mutation status. **A** Kaplan-Meier survival analysis comparing overall survival between TLE1 high and low expression groups in the EGFR mutation subgroup in the TCGA_LUAD cohort. **B** Kaplan-Meier survival analysis comparing overall survival between TLE1 high and low expression groups in the EGFR-wildtype subgroup in the TCGA_LUAD cohort.

Supplementary Material 5. Table S1. A list of 718 histone modification-related genes.

Supplementary Material 6. Table S2. A list of 15 epithelial-mesenchymal transition (EMT)-related genes.

Supplementary Material 7. Table S3. A list of 3833 genes significantly associated with LUAD prognosis.

Supplementary Material 8. Table S4. GO and KEGG enrichment analysis of 71 osimertinib resistance-related genes in LUAD ranked by *p* value.

Supplementary Material 9. Table S5. A list of 506 differential expression genes.

Supplementary Material 10. Table S6. GO and KEGG enrichment analysis of 506 TLE1-related genes in LUAD ranked by p value.

Supplementary Material 11. Table S7. Specific rankings and scores of the 100 hub genes.

Supplementary Material 12. Table S8 Complete drug sensitivity analysis results.

Supplementary Material 13.

## Data Availability

Data supporting the conclusion of this article are openly available in The Cancer Genome Atlas (TCGA) database (https://portal.gdc.cancer.gov/) and Gene Expression Omnibus (GEO database; https://www.ncbi.nlm.nih.gov/geo/).
