# Supplementary figures and images for: TLE1 as a key regulator of osimertinib resistance and EMT in lung adenocarcinoma: implications for prognosis and immunotherapy response
Source: Hereditas. 2026 May 28;163:84. doi: 10.1186/s41065-026-00690-x (PMC13425785; doi:10.1186/s41065-026-00690-x)

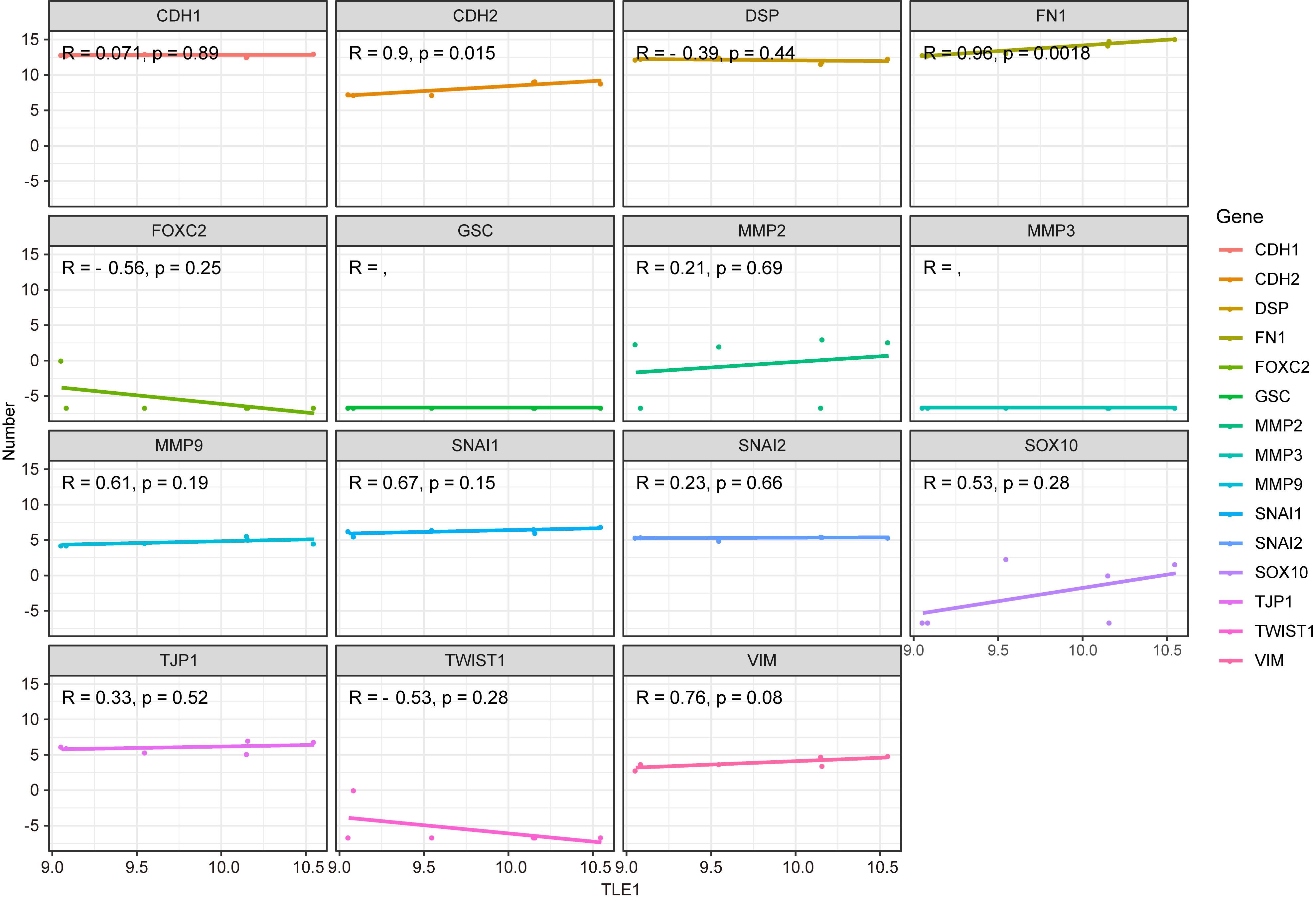

Supplement: Supplementary file 1 — Supplementary Material 1. Figure S1 Correlation analysis of TLE1 expression level and 15 EMT-related markers in the GSE222820 cohort. [file 41065_2026_690_MOESM1_ESM.tif]

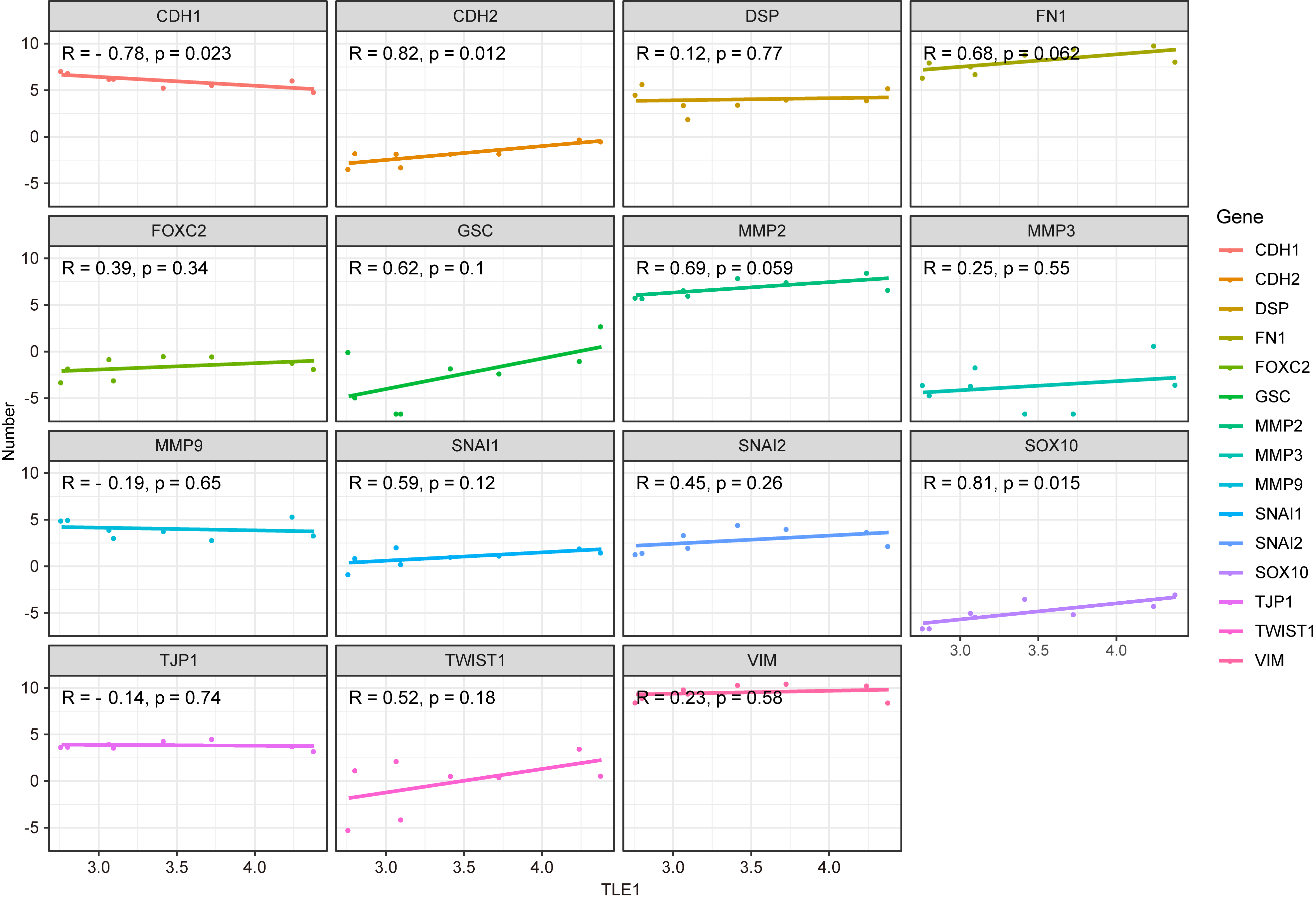

Supplement: Supplementary file 2 — Supplementary Material 2. Figure S2 Correlation analysis of TLE1 expression level and 15 EMT-related markers in the GSE253742 cohort. [file 41065_2026_690_MOESM2_ESM.tif]

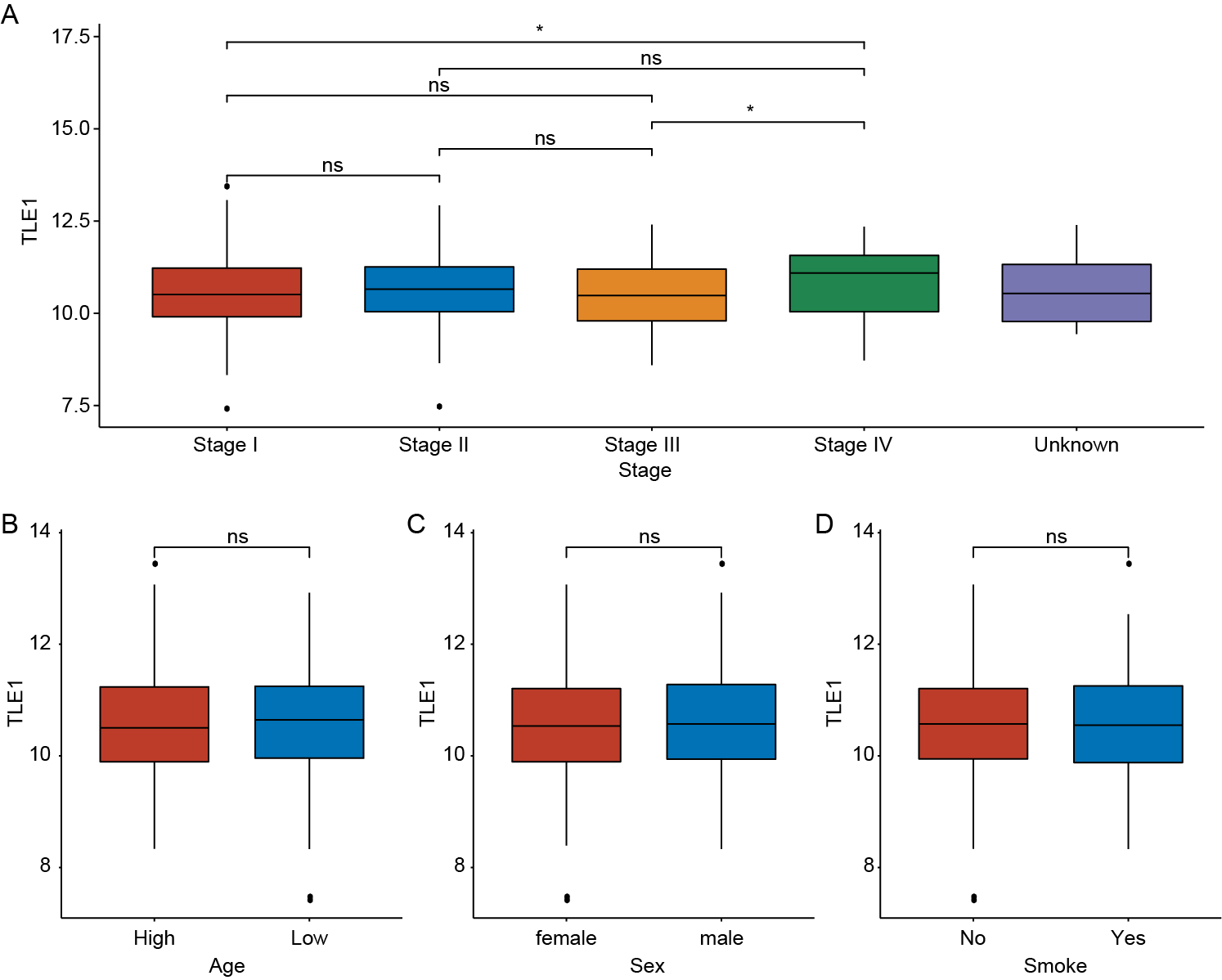

Supplement: Supplementary file 3 — Supplementary Material 3. Figure S3 Clinicopathological parameter relevance analysis. A-D Box diagram illustrate the relationships between TLE1 expression and various clinical pathological features: Stage (A), Age (B), Gender (C) and Smoking status (D) in the TCGA_LUAD cohort. [file 41065_2026_690_MOESM3_ESM.tif]

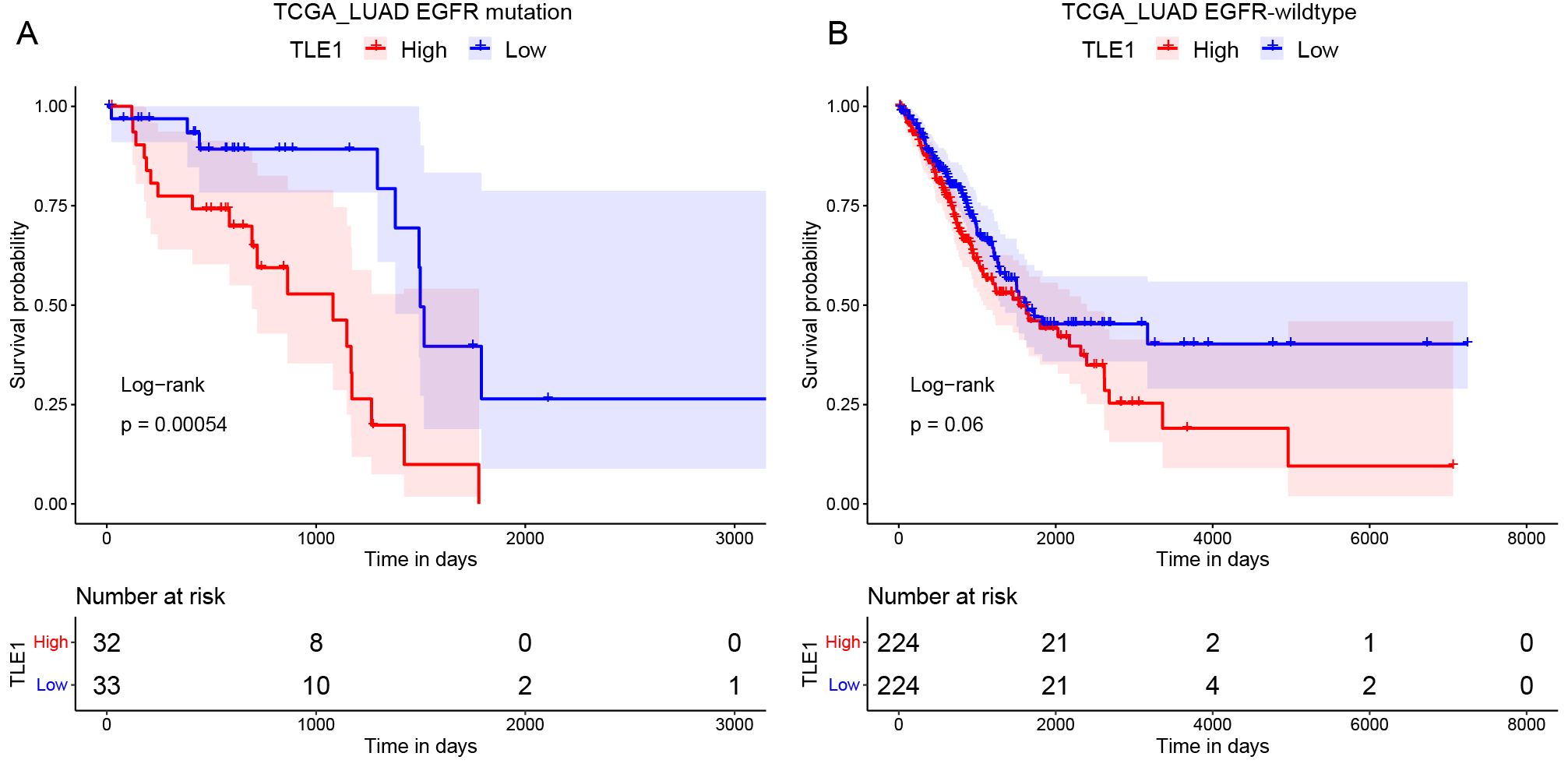

Supplement: Supplementary file 4 — Supplementary Material 4. Figure S4 Kaplan-Meier survival analysis of TLE1 expression in LUAD patients stratified by EGFR mutation status. A Kaplan-Meier survival analysis comparing overall survival between TLE1 high and low expression groups in the EGFR mutation subgroup in the TCGA_LUAD cohort. B Kaplan-Meier survival analysis comparing overall survival between TLE1 high and low expression groups in the EGFR-wildtype subgroup in the TCGA_LUAD cohort. [file 41065_2026_690_MOESM4_ESM.tif]
