# Supplementary material for: TLE1 as a key regulator of osimertinib resistance and EMT in lung adenocarcinoma: implications for prognosis and immunotherapy response
Source: Hereditas. 2026 May 28;163:84. doi: 10.1186/s41065-026-00690-x (PMC13425785; doi:10.1186/s41065-026-00690-x)
Supplement: Supplementary file 6 — Supplementary Material 6. Table S2. A list of 15 epithelial-mesenchymal transition (EMT)-related genes. [file 41065_2026_690_MOESM6_ESM.docx]

**Table S2. A list of 15 epithelial-mesenchymal transition (EMT)-related genes.**

| “Mesenchymal” markers | “Epithelial” markers |
| --- | --- |
| FOXC2 | CDH1 |
| CDH2 | DSP |
| FN1 | TJP1 |
| GSC |  |
| MMP2 |  |
| MMP3 |  |
| MMP9 |  |
| SNAI1 |  |
| SNAI2 |  |
| SOX10 |  |
| TWISTI |  |
| VIM |  |
