# Supplementary material for: TLE1 as a key regulator of osimertinib resistance and EMT in lung adenocarcinoma: implications for prognosis and immunotherapy response
Source: Hereditas. 2026 May 28;163:84. doi: 10.1186/s41065-026-00690-x (PMC13425785; doi:10.1186/s41065-026-00690-x)

Figure 8C
（Raw Data）

Bright-field image of the membrane（General view, no specific band development yet）

Film exposure result of target bands（TLE1 and β-actin for samples, clear bands at specific molecular weight）

Bright-field image of the membrane with target bands visualized（ at ~70-100 or 40-50 kd range ）

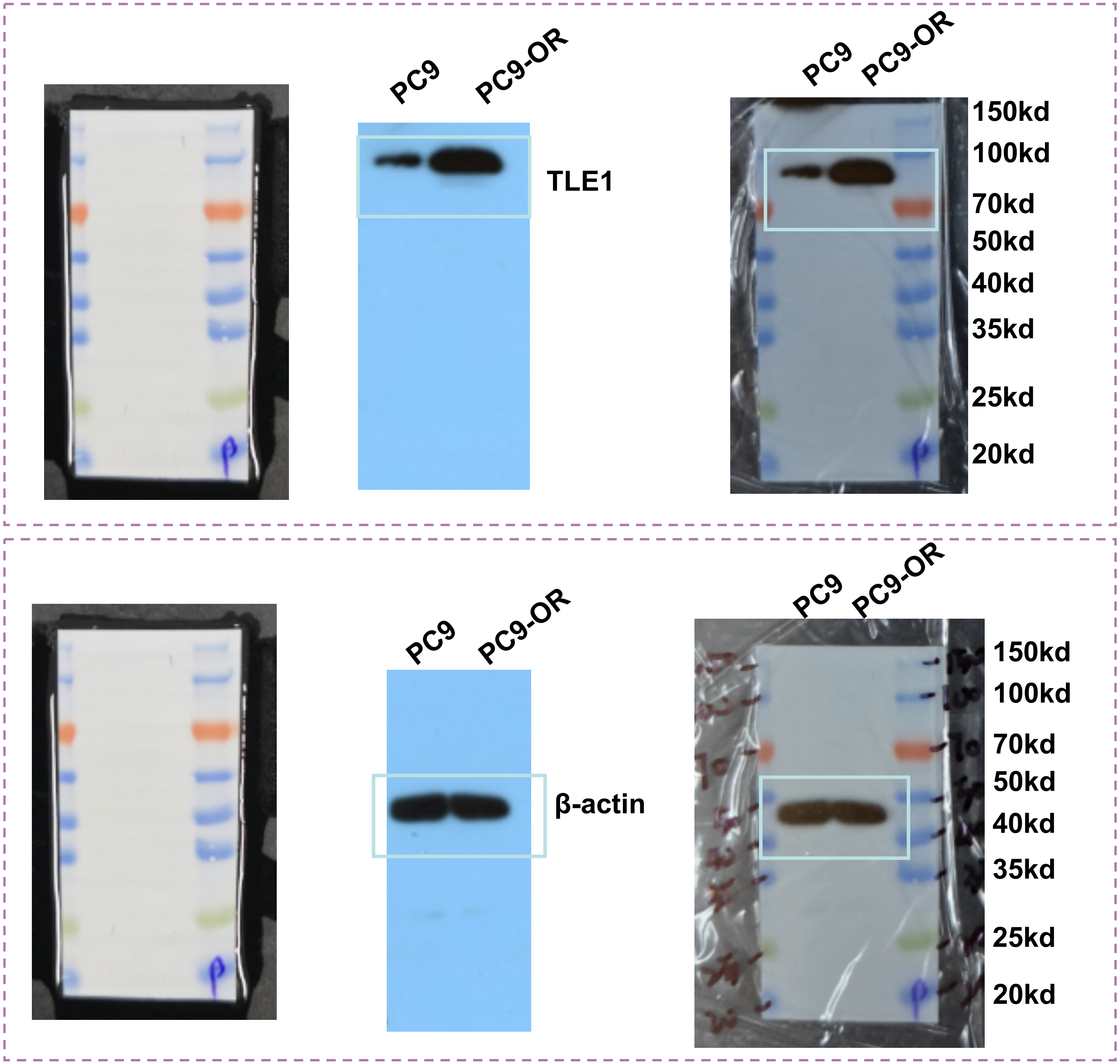

Figure 8E
（Raw Data）

Bright-field image of the membrane（General view, no specific band development yet）

Film exposure result of target bands（TLE1 and β-actin for samples, clear bands at specific molecular weight ）

Bright-field image of the membrane with target bands visualized（ at ~70-100 or 40-50 kd range ）

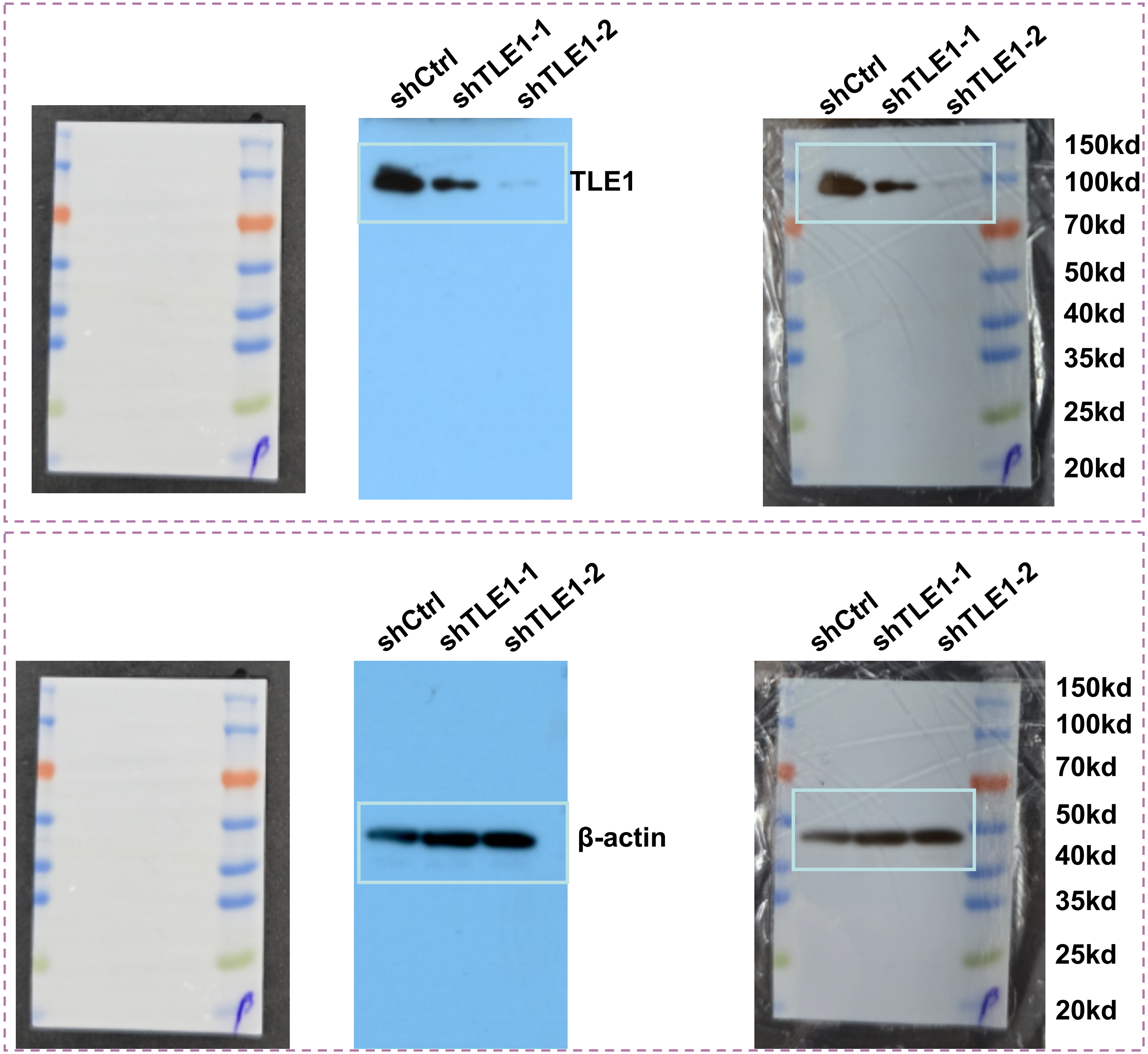

Supplement: Supplementary file 13 — Supplementary Material 13. [file 41065_2026_690_MOESM13_ESM.pdf]
